# Maintaining the Quality and Storage Life of Button Mushrooms *(Agaricus bisporus*) with Gum, Agar, Sodium Alginate, Egg White Protein, and Lecithin Coating

**DOI:** 10.3390/jof7080614

**Published:** 2021-07-29

**Authors:** Seyda Cavusoglu, Yusuf Uzun, Nurettin Yilmaz, Sezai Ercisli, Erkan Eren, Halina Ekiert, Hosam O. Elansary, Agnieszka Szopa

**Affiliations:** 1 Department of Horticulture, Faculty of Agriculture, Van Yuzuncu Yil University, Van 65080, Turkey; scavusoglu@yyu.edu.tr (S.C.); nrtnylmz47@gmail.com (N.Y.); 2 Department of Pharmaceutical Sciences, Faculty of Pharmacy, Van Yüzüncü Yıl University, Van 65080, Turkey; y.uzun@yyu.edu.tr; 3 Department of Horticulture, Faculty of Agriculture, Atatürk University, Erzurum 25240, Turkey; 4 Bergama Technical and Business College, Mushroom Programme, Ege University, Izmir 35700, Turkey; erkan.eren@ege.edu.tr; 5 Department of Pharmaceutical Botany, Faculty of Pharmacy, Medical College, Jagiellonian University, Medyczna 9, 30-688 Kraków, Poland; halina.ekiert@uj.edu.pl (H.E.); a.szopa@uj.edu.pl (A.S.); 6 Plant Production Department, College of Food and Agricultural Sciences, King Saud University, Riyadh 11451, Saudi Arabia; helansary@ksu.edu.sa

**Keywords:** *Agaricus bisporus*, edible coatings, external ethylene, respiration rate, storage, quality

## Abstract

Button mushrooms have a very short shelf life after harvesting and are sensitive to mechanical damage and browning. This can be a severe problem in enlarging the market and the long-distance exportation of this product. In this respect, edible coatings could be an alternative treatment to extend the shelf life of button mushrooms, maintaining their quality during long-term storage. The aim of this study was to investigate the impact of gum, agar, sodium alginate, egg white protein, and lecithin on the postharvest weight loss, color, browning, respiration rate, ethylene production, and storage life of button mushrooms. The results showed that the above-mentioned edible coatings are a promising way to extend the life and maintain the quality of button mushrooms. Significant differences (*p* < 0.05) were observed between the control and edible coating-treated samples in all parameters. Sodium alginate and gum were more effective in preventing weight loss, coloring, and browning than other edible coatings. On the other hand, the respiration rate and ethylene production were more suppressed by the agar and lecithin coatings compared to the others. In conclusion, it can be recommended that the above-mentioned edible coatings could be used as novel coatings in commercial treatments for maintaining the quality of button mushrooms during a long-term storage period.

## 1. Introduction

Button mushrooms (*Agaricus bisporus*) have an important place in the world trade of fresh produce because they contain important bioactive compounds such as vitamins, minerals, polyphenolics, and flavonoids preferred by most consumers [1,2]. Since button mushrooms have a very short shelf life after harvesting, special protection techniques are required to maintain their quality and freshness.

There are various treatments for extending the shelf life of mushrooms, including washing them with citric acid, ethylenediaminetetraacetic acid (EDTA), hydrogen peroxide, and sodium hypochlorite. It has been reported that hydrogen peroxide, citric acid [3,4,5], methyl jasmonate [6], essential oils [7], sodium metabisulfite [8], alginate [9], natamycin [10], 4-methoxy cinnamic acid [11], high-pressure argon and ultrasound [12], glycine betaine [13], CaCl_2_ [5], and coatings [14] have significant positive effects on mushroom preservation. Among these treatments, edible coatings are traditionally used to enhance postharvest food appearance and preservation, as edible coatings provide products with a sheen and make them more attractive to consumers [15]. Moreover, they maintain the phytochemical (antioxidants, phenolics, and color) and physicochemical (weight loss, respiration rate, and ethylene production) properties for a longer period, and some edible coatings act as a natural antimicrobial and antifungal compound in many fruits and vegetables [16].

Edible coatings consist of polysaccharides, proteins, and lipids made from various agricultural products and food processing wastes and byproducts [17,18]. Polysaccharide coatings are hydrophilic and include chitosan, pectin, carrageenan, cellulose derivatives, starch derivatives, alginate, agar, and gums [19]. Proteins are also hydrophilic and include corn zein, wheat gluten, peanut, soy, collagen, gelatin, whey, casein, and egg white protein [20,21]. Furthermore, lecithin has been used as an emulsifier to dissolve in coatings containing oil [22]. Edible coatings generally act as a barrier to gas exchange properties and thus prolong the storage life of fruit and vegetables [23,24].

In recent years, the increasing consumption of fresh produce worldwide has led to the necessity for alternative biocontrol methods with a high efficiency, with a low residue rate, that are non-toxic, that are environmentally and economically friendly, and which do not threaten human health. Edible coatings applied as a thin layer on the product’s surface are biodegradable materials that have no adverse effects on human health and are environmentally and economically friendly. In this respect, edible coating materials are promising treatments for extending the commercial storage life of fresh fruit and vegetables. To our knowledge, there have been few published studies about the effect of gum, agar, and sodium alginate, and no studies about the effect of egg white protein (EWP) and lecithin on extending the storage life of button mushrooms. Therefore, the aim of this study was to figure out an approach that could be used to extend the storage life of button mushrooms and examine the effects of the above-mentioned edible coatings on the physicochemical (weight loss, respiration rate, and ethylene production) and sensory qualities (color and browning) of button mushrooms with modified atmosphere packaging.

## 2. Materials and Methods

### 2.1. Materials

Composts were purchased from a commercial company (Yiğit Mantar, Ankara, Turkey). Mushrooms were grown in rooms where growing conditions could be controlled, belonging to Van Yuzuncu Yil University Mushroom Research and Treatment Center. After mushrooms were successfully grown and harvested, mushrooms of uniform size, without any browning symptoms, and free from mechanical damage were selected for the experiment.

### 2.2. Preparation of Edible Coating and Treatments

All harvested samples were first exposed to a Vilber Lourmat UV-C lamb with 254 nm (0.25 kJ/m^2^) on all surfaces for 2 min from a distance of 20 cm for sterilization. The edible coatings were purchased from a commercial company. Edible coatings made up of gum (2.75 g), agar (2.75 g), sodium alginate (2.5 g), EWP (5 g), and lecithin (5 g) were prepared by dissolving them in 500 mL of distilled water. The pH of the coating solutions was adjusted to 6.0. After preparing the edible coatings, the mushrooms were randomly divided into six groups. While the control group was dipped in distilled water, the others were dipped for 2 min at 20 °C in the previously prepared coating solutions. After treatment, all samples were dried at room temperature (20 °C). Later, the samples were placed on foam plates (300 g each pack) and covered with stretch film (eight microns) for 15 days at a temperature of 4 °C and a 90–95% relative humidity (RH).

The quality of the samples was analyzed at 0, 5, 10, and 15 days of storage, and a list of quality parameters including weight loss, color, browning index, respiration rate, ethylene production, and the concentration of O_2_ and CO_2_ inside the packages were measured at selected days.

### 2.3. Weight Loss (WL)

Weight loss was measured by a precision scale at 5-day intervals and calculated as a percentage of the initial weight.

### 2.4. Color and Browning Index (BI)

The color of the mushroom caps (10 samples for each replicate) was measured by a colorimeter (Minolta CR-400; Osaka, Japan) in L*, *C°,* and *h°* color space. The browning index (BI) was calculated as described by Karimirad et al. [25]:

BI = [100(X − 0.31)]/0.17,

where X = (a* − 1.75 L*)/(5.645 L* + a* − 3.012 b*).

### 2.5. Respiration Rate (RR) and Ethylene Production (EP)

In order to determine the respiration rate, the mushrooms (300 g) were kept in closed 2 L jars for 2 h, and the carbon dioxide (CO_2_) emission of the mushrooms was then measured with the Quantek Headspace Gas Analyzer GS3/L (Grafton, MA, USA). The respiration rate values are expressed as mL CO_2_ kg^−1^·h^−1^ [26]. The ethylene production of samples was measured according to the methods of Çavuşoğlu [26]. The ethylene production is expressed as mL C_2_H_4_ kg^−1^·h^−1^.

The oxygen and carbon dioxide concentrations in the packages were measured by the Headspace Gas Analyzer GS3/L.

### 2.6. Statistical Analysis

This experiment was carried out as a completely randomized experimental design with three replications and each package was evaluated after one replication. Descriptive statistics for the studied variables were presented as mean and the Standard Error of the Mean (SEM). Two-way Factorial ANOVA was performed on the data. Treatments with different edible coatings and storage periods were considered as factors. Duncans’ Multiple Range Test comparisons were also used to identify different levels of treatment and storage factors. In addition, Pearson’s correlation coefficients between the measured parameters were found. The statistical significance level was considered as 5% and the SPSS (ver: 20) statistical program was used for all statistical computations.

## 3. Results

### 3.1. Weight Loss

Weight loss increased in all samples, regardless of treatment, during the 15-day storage period. Furthermore, weight loss was significantly (*p* < 0.05) lower in the edible coating-treated samples than in the uncoated samples. Significant differences (*p* < 0.05) were observed between the storage periods (Table 1).

### 3.2. Color and Browning Index (BI)

The level of L* values decreased in all samples during the storage period. However, the sample treated with edible coatings resulted in higher values of L* at all the sampling time intervals compared to the uncoated samples. The highest values of L* were 81.71 and 81.24 for the gum-treated samples and EWP-treated samples, respectively. L* values were significantly (*p* < 0.05) higher in the edible coating-treated samples than in uncoated samples. In addition, there were significant (*p* < 0.05) differences among the storage periods (Table 2).

The value of *C°* steadily increased in all samples throughout storage. However, the highest value of *C°* was 24.28 in the control samples after 15 days of storage. There was a significant (*p* < 0.05) difference among treatments. Significant differences (*p* < 0.05) were also observed among storage periods (Table 2).

The highest value of *h°* was observed in control samples with a value of 92.93, while the lowest value was observed in EWP-treated samples with a value of 95.12 at the end of the storage period. Significant differences (*p* < 0.05) were observed between the control and edible coating-treated samples. Significant differences (*p* < 0.05) were observed among storage periods (Table 2).

The browning index (BI) in both edible coating-treated and control samples showed a trend of increase during storage. However, BI showed a lower trend in the samples treated with an edible coating compared with uncoated samples. The lowest values of BI were 30.06 and 31.19 for sodium alginate-treated and gum-treated samples, respectively. BI was significantly (*p* < 0.05) lower in the edible coating-treated samples than that in the uncoated samples. There were significant (*p* < 0.05) differences among the storage periods (Table 2).

### 3.3. Respiration Rate (RR) and Ethylene Production (EP)

Ethylene production decreased sharply within the initial 5 d of storage in all samples. However, samples treated with an edible coating suppressed ethylene production more than the control samples. Ethylene production was significantly (*p* < 0.05) lower in the edible coating-treated samples compared with the uncoated samples. Significant differences (*p* < 0.05) were observed among the storage periods (Table 3).

The respiration rate reached a peak on the fifth day and subsequently decreased in all samples. Treatment with an edible coating markedly decreased the respiration rate compared to the uncoated samples. The respiration rate was significantly (*p* < 0.05) lower in the edible coating-treated samples than in the uncoated samples. There were significant (*p* < 0.05) differences among the storage periods (Table 3).

### 3.4. Oxygen and Carbon Dioxide Concentrations in the Packages

On the fifth day of storage, the CO_2_ levels increased dramatically inside the package, while the O_2_ levels reduced. At all the sampling time intervals, lower CO_2_ levels were found in samples treated with an edible coating; furthermore, higher O_2_ levels were found in samples treated with an edible coating compared to the control samples. The O_2_ levels were significantly (*p* < 0.05) lower in the edible coating-treated samples, but the CO_2_ levels were significantly (*p* < 0.05) higher in the edible coating-treated samples compared with the uncoated samples. There were significant (*p* < 0.05) differences among the storage periods in both the O_2_ and CO_2_ levels (Table 3).

## 4. Discussion

Weight loss indicating the quality and freshness of mushrooms is mainly related to the respiration rate and moisture evaporation through the mushroom’s surface. If weight loss in mushrooms is more than 4–6%, they become unmarketable because high levels of weight loss lead to losses of quality and are related to visible signs of wilting or shrinkage [27]. It was reported in different studies that weight loss can be reduced by treatment with agar-agar [28], sodium alginate [29], chitosan, and guar gum [17]. We obtained similar results from the current study where the edible coating-treated samples showed lower weight loss than uncoated samples. In our study, disinfection was carried out with UV-C, and this gave more positive results in terms of weight loss than the application of NaClO_2_ [30].

The color of button mushrooms is probably the biggest indicator of quality for consumers because it is associated with the age of the mushrooms and is used as an indicator to determine the shelf life and freshness. In cases where the last value of L* is lower than 80 in button mushrooms, wholesalers may not class them as commercially acceptable [7]. Mushrooms are initially white when harvested but, as the storage days continue, the discoloration on the cap increases because of enzymatic reactions [31]. The enzymes which are responsible for browning react with the substrate, and brown pigmentation occurs. If there is no more substrate during the storage period, the enzymatic reaction decreases and the evolution of the brown pigmentation ceases [32]. Browning occurs due to two precise mechanisms of phenol oxidation by the activation of tyrosinase or spontaneous oxidation [32,33]. Enzymatic browning results from the PPO-catalyzed oxidation of phenolic substrates to quinones, which exposes their reaction to dark pigments knows as melanins. The main PPO enzyme responsible for browning in mushrooms appears to be tyrosinase [32,34]. It was reported in earlier studies that discoloration and browning were delayed in fresh-cut Chinese water chestnut and *Agaricus bisporus* with the treatment of chitosan [35], chitosan nanoparticles containing Cuminum cyminum oil [25], and A. vera gel alone/combined with basil essential oil [36]. In the present study, the edible coating-treated samples delayed discoloration and browning. A positive correlation was found between BI and weight loss (Table 4). Furthermore, a similar correlation was reported in litchi fruit [37] and button mushrooms [36].

During the respiration process, the O_2_ level affects the metabolic process of respiration rate and the senescence of fruits and vegetables depends on the respiration rate. Therefore, a lower respiration rate plays a vital role in extending the life of fruit and vegetables during the postharvest period [38,39,40,41]. The treatments of coating the product surface [42] and modifying the atmosphere packaging [26,43,44] could be used as alternative ways to limit gas exchange properties, reducing the O_2_ available. It has been reported that treating fruit with an edible coating suppresses the respiration rate in button mushrooms [36]. Moreover, treating fruit with an edible coating suppresses the respiration rate and decreases ethylene production in various fruits, including kiwifruits [45], peaches [46], and avocados [47]. In the present study, we supported the aforementioned studies that the edible coating of mushrooms slows down the respiration rate and ethylene production compared to control fruit.

## 5. Conclusions

In conclusion, the treatment of an edible coating significantly delayed senescence and maintained the quality of button mushrooms during the storage period. Although no differences were found among the edible coating materials in terms of the investigated parameters, some were relatively more effective than others. For example, sodium alginate and gum were more effective at preventing weight loss, coloring, and browning than the other edible coatings. Furthermore, the respiration rate and ethylene production were more suppressed by the agar and lecithin coatings compared to the others. Therefore, it can be recommended that the above-mentioned edible coatings could be used as novel coatings in commercial treatments to maintain the quality of button mushrooms during a long-term storage period.

## Figures and Tables

**Table 1:** The changes in weight loss during the storage of button mushrooms during 15 d at 4 °C. Data are presented as means ± SEM.

Weight Loss (%)	Storage Period (d)				
Treatment	0	5	10	15	Means
Control	0.00 ± 0.00	2.75 ± 0.29 ^A^	5.22 ± 0.64 ^A^	7.68 ± 0.55 ^A^	3.91 ± 0.88 ^A^
EWP	0.00 ± 0.00	1.24 ± 0.21 ^B^	2.88 ± 0.18 ^B^	4.07 ± 0.18 ^B^	2.05 ± 0.47 ^B^
Lecithin	0.00 ± 0.00	1.36 ± 0.17 ^B^	2.78 ± 0.18 ^B^	4.29 ± 0.16 ^B^	2.11 ± 0.49 ^B^
Gum	0.00 ± 0.00	0.96 ± 0.46 ^B^	2.77 ± 1.00 ^B^	4.60 ± 0.96 ^B^	2.08 ± 0.62 ^B^
Agar	0.00 ± 0.00	1.73 ± 0.56 ^AB^	2.44 ± 0.12 ^B^	3.86 ± 0.37 ^B^	2.01 ± 0.44 ^B^
Sodium alginate	0.00 ± 0.00	1.53 ± 0.31 ^B^	2.43 ± 0.21 ^B^	3.41 ± 0.27 ^B^	1.84 ± 0.39 ^B^
Means	0.00 ± 0.00 ^d^	1.60 ± 0.18 ^c^	3.09 ± 0.29 ^b^	4.65 ± 0.38 ^a^	
Significant effects; *p*^treatment^ = 0.11		*p*^storage^ = 0.01		*p*^treatment^ × *p*^storage^ = 0.01	

Differences among storage periods are shown with small letters (*p* < 0.05), differences among treatments are shown with capital letters (*p* < 0.05).

**Table 2:** The changes in L*, *C°*, *hue,* and browning index (BI) during the storage of button mushrooms over 15 d at 4 °C. Data are presented as means ± SEM.

L*	Storage Period (d)				
Treatment	0	5	10	15	Means
Control	87.36 ± 0.68	81.54 ± 1.23 ^B^	79.25 ± 0.94 ^C^	74.53 ± 0.45 ^C^	80.67 ± 1.44 ^B^
EWP	87.36 ± 0.68	85.98 ± 0.18 ^A^	84.60 ± 0.44 ^A^	81.24 ± 0.53 ^A^	84.79 ± 0.72 ^A^
Lecithin	87.36 ± 0.68	83.72 ± 1.22 ^AB^	81.73 ± 0.79 ^B^	79.00 ± 0.24 ^B^	82.95 ± 0.98 ^AB^
Gum	87.36 ± 0.68	85.81 ± 0.85 ^A^	83.40 ± 0.31 ^AB^	81.71 ± 0.54 ^A^	84.57 ± 0.71 ^A^
Agar	87.36 ± 0.68	84.26 ± 0.78 ^AB^	82.25 ± 0.65 ^B^	81.10 ± 0.50 ^A^	83.74 ± 0.77 ^A^
Sodium alginate	87.36 ± 0.68	85.39 ± 0.20 ^A^	82.97 ± 0.18 ^AB^	81.08 ± 0.97 ^A^	84.20 ± 0.76 ^A^
Means	87.36 ± 0.99 ^a^	84.45 ± 2.01 ^b^	82.37 ± 1.93 ^c^	79.78 ± 2.71 ^d^	
Significant effects; *p*^treatment^ = 0.02			*p*^storage^ = 0.01	*p*^treatment^ × *p*^storage^ = 0.01	
*C°*					
Control	15.25 ± 0.64	19.45 ± 0.82 ^AB^	22.65 ± 0.66 ^A^	24.28 ± 0.47^A^	20.41 ± 1.08
EWP	15.25 ± 0.64	18.13 ± 0.50 ^BC^	19.03 ± 0.27 ^B^	20.48 ± 0.39 ^C^	18.22 ± 0.61
Lecithin	15.25 ± 0.64	20.62 ± 0.13 ^A^	21.44 ± 0.19 ^A^	22.16 ± 0.76 ^B^	19.87 ± 0.85
Gum	15.25 ± 0.64	17.93 ± 0.16 ^BC^	19.53 ± 0.34 ^B^	20.84 ± 0.40 ^BC^	18.39 ± 0.65
Agar	15.25 ± 0.64	17.38 ± 0.54 ^C^	19.52 ± 0.42 ^B^	21.40 ± 0.47 ^BC^	18.39 ± 0.73
Sodium alginate	15.25 ± 0.64	18.05 ± 0.82 ^BC^	18.91 ± 0.85 ^B^	21.55 ± 0.08 ^BC^	18.44 ± 0.74
Means	15.25 ± 0.92 ^d^	18.59 ± 1.40 ^c^	20.18 ± 1.60 ^b^	21.79 ± 1.45 ^a^	
Significant effects; *p*^treatment^ = 0.23			*p*^storage^ = 0.01	*p*^treatment^ × *p*^storage^ = 0.01	
*hue*					
Control	84.46 ±0.13	85.78 ± 0.42 ^A^	88.65 ± 1.02 ^A^	92.93 ± 1.40 ^A^	87.96 ± 1.05 ^B^
EWP	84.46 ± 0.13	85.17 ± 0.24 ^AB^	84.89 ± 0.24 ^B^	85.12 ± 0.61 ^B^	84.91 ± 0.17 ^A^
Lecithin	84.46 ± 0.13	84.70 ± 0.46 ^AB^	86.09 ± 0.47 ^B^	85.20 ± 0.48 ^B^	85.11 ± 0.26 ^A^
Gum	84.46 ± 0.13	85.47 ± 0.50 ^A^	84.97 ± 0.75 ^B^	85.43 ± 0.54 ^B^	85.08 ± 0.26 ^A^
Agar	84.46 ± 0.13	84.00 ± 0.20 ^B^	85.71 ± 1.46 ^B^	83.87 ± 0.11 ^B^	84.51 ± 0.38 ^A^
Sodium alginate	84.46 ± 0.13	84.94 ± 0.18 ^AB^	85.06 ± 0.05 ^B^	85.54 ± 0.23 ^B^	85.00 ± 0.14 ^A^
Means	84.46 ± 0.20 ^c^	85.01 ± 0.79 ^bc^	85.89 ± 1.80 ^ab^	86.35 ±3.24 ^a^	
Significant effects; *p*^treatment^ = 0.01			*p*^storage^ = 0.01	*p*^treatment^ × *p*^storage^ = 0.01	
BI					
Control	19.79 ± 0.88	28.30 ± 1.73 ^AB^	38.30 ± 2.15 ^A^	46.24 ± 0.98 ^A^	33.16 ± 3.08 ^A^
EWP	19.79 ± 0.88	24.44 ± 1.02 ^B^	27.33 ± 1.14 ^BC^	31.47 ± 1.00 ^BC^	25.76 ± 1.35 ^B^
Lecithin	19.79 ± 0.88	29.27 ± 0.99 ^A^	31.59 ± 0.89 ^B^	33.81 ± 0.86 ^B^	28.61 ± 1.66 ^AB^
Gum	19.79 ± 0.88	26.68 ± 1.74 ^AB^	29.06 ± 0.40 ^BC^	31.19 ± 0.20 ^BC^	26.68 ± 1.36 ^B^
Agar	19.79 ± 0.88	25.78 ± 1.15 ^AB^	27.54 ± 1.53 ^BC^	32.62 ± 0.92 ^BC^	26.43 ± 1.47 ^B^
Sodium alginate	19.79 ± 0.88	24.25 ± 1.27 ^B^	26.31 ± 1.43 ^C^	30.06 ± 1.29 ^C^	25.10 ± 1.24 ^B^
Means	19.79 ± 1.28 ^d^	26.45 ± 2.74 ^c^	30.02 ± 4.63 ^b^	34.23 ± 5.82 ^a^	
Significant effects; *p*^treatment^ = 0.02			*p*^storage^ = 0.01	*p*^treatment^ × *p*^storage^ = 0.01	

Differences among storage periods are shown with small letters (*p* < 0.05), differences among treatments are shown with capital letters (*p* < 0.05).

**Table 3:** The changes in respiration rate, ethylene production, O_2_, and CO_2_ inside the packages during the storage of button mushrooms during 15 d at 4 °C. Data are presented as means ± SEM.

Ethylene	Storage Period (d)				
Treatment	0	5	10	15	Means
Control	2.46 ± 0.06	1.88 ± 0.01 ^A^	1.80 ± 0.01 ^A^	1.54 ± 0.01 ^A^	1.92 ± 0.10 ^A^
EWP	2.46 ± 0.06	0.65 ± 0.03 ^D^	0.91 ± 0.01 ^E^	0.95 ± 0.01 ^B^	1.24 ± 0.21 ^B^
Lecithin	2.46 ± 0.06	0.85 ± 0.04 ^C^	0.88 ± 0.01 ^E^	0.77 ± 0.01 ^D^	1.24 ± 0.21 ^B^
Gum	2.46 ± 0.06	1.23 ± 0.01 ^B^	1.47 ± 0.02 ^B^	0.97 ± 0.01 ^B^	1.53 ± 0.17 ^AB^
Agar	2.46 ± 0.06	0.81 ± 0.02 ^C^	1.10 ± 0.01 ^D^	0.67 ± 0.01 ^E^	1.26 ± 0.21 ^B^
Sodium alginate	2.46 ± 0.06	0.69 ± 0.01 ^D^	1.22 ± 0.01 ^C^	0.86 ± 0.02 ^C^	1.31 ± 0.21 ^B^
Means	2.46 ± 0.09 ^a^	1.02 ± 0.44 ^c^	1.23 ± 0.33 ^b^	0.96 ± 0.29 ^c^	
Significant effects; *p*^treatment^ = 0.09			*p*^storage^ = 0.01	*p*^treatment^ × *p*^storage^ = 0.01	
Respiration rate					
Control	95.94 ± 1.60	117.52 ± 3.80 ^A^	96.59 ± 1.63 ^A^	80.66 ± 1.21 ^A^	97.68 ± 4.07 ^A^
EWP	95.94 ± 1.60	104.38 ± 1.29 ^BC^	86.57 ± 0.91 ^B^	68.08 ± 1.15 ^B^	88.74 ± 4.10 ^AB^
Lecithin	95.94 ± 1.60	101.88 ± 2.05 ^C^	71.13 ± 0.98 ^D^	62.80 ± 1.41 ^C^	82.94 ± 4.98 ^AB^
Gum	95.94 ± 1.60	104.86 ± 0.59 ^BC^	78.52 ± 1.56 ^C^	69.26 ± 0.57 ^B^	87.15 ± 4.26 ^AB^
Agar	95.94 ± 1.60	102.57 ± 0.75 ^C^	70.74 ± 1.61 ^D^	48.82 ± 1.36 ^D^	79.52 ± 6.46 ^B^
Sodium alginate	95.94 ± 1.60	109.92 ± 2.57 ^B^	89.83 ± 2.09 ^B^	66.56 ± 1.17 ^B^	90.56 ± 4.79 ^AB^
Means	95.94 ± 2.32 ^b^	106.86 ± 6.40 ^a^	82.23 ± 10.11 ^c^	66.03 ± 9.87 ^d^	
	Significant effects; *p*^treatment^ = 0.14		*p*^storage^ = 0.01	*p*^treatment^ × *p*^storage^ = 0.01	
O_2_					
Control	20.90 ± 0.00	16.03 ± 0.09 ^A^	15.10 ± 0.15 ^AB^	14.77 ± 0.07 ^A^	16.70 ± 0.75
EWP	20.90 ± 0.00	15.00 ± 0.06 ^B^	15.00 ± 0.30 ^ABC^	14.53 ± 0.26 ^AB^	16.36 ± 0.80
Lecithin	20.90 ± 0.00	16.00 ± 0.47 ^A^	15.73 ± 0.18 ^A^	14.00 ± 0.17 ^ABC^	16.66 ± 0.78
Gum	20.90 ± 0.00	14.57 ± 0.15 ^BC^	14.40 ± 0.15 ^BCD^	13.50 ± 0.17 ^C^	15.84 ± 0.89
Agar	20.90 ± 0.00	13.90 ± 0.40 ^C^	13.90 ± 0.42 ^A^	13.73 ± 0.37 ^BC^	15.61 ± 0.93
Sodium alginate	20.90 ± 0.00	14.33 ± 0.44 ^BC^	14.03 ± 0.49 ^CD^	13.73 ± 0.53 ^BC^	15.75 ± 0.92
Means	20.90 ± 0.00 ^a^	14.97 ± 0.95 ^b^	14.69 ± 0.80 ^b^	14.04 ±0.65 ^c^	
Significant effects; *p*^treatment^ = 0.89			*p*^storage^ = 0.01	*p*^treatment^ × *p*^storage^ = 0.01	
CO_2_					
Control	0.30 ± 0.00	1.70 ± 0.06 ^E^	1.90 ± 0.06 ^C^	2.00 ± 0.06 ^B^	1.48 ± 0.21
EWP	0.30 ± 0.00	2.07 ± 0.03 ^CD^	2.10 ± 0.06 ^BC^	2.40 ± 0.06 ^A^	1.72 ± 0.25
Lecithin	0.30 ± 0.00	2.40 ± 0.06 ^AB^	2.37 ± 0.12 ^B^	2.57 ± 0.22 ^A^	1.91 ± 0.29
Gum	0.30 ± 0.00	1.83 ± 0.03 ^DE^	2.07 ± 0.09 ^C^	2.50 ± 0.12 ^A^	1.68 ± 0.25
Agar	0.30 ± 0.00	2.60 ± 0.06 ^A^	2.50 ± 0.10 ^A^	2.67 ± 0.09 ^A^	2.02 ± 0.30
Sodium alginate	0.30 ± 0.00	2.27 ± 0.17 ^BC^	2.33 ± 0.03 ^AB^	2.50 ± 0.12 ^A^	1.85 ± 0.27
Means	0.30 ± 0.00 ^c^	2.14 ± 0.34 ^b^	2.21 ± 0.24 ^b^	2.44 ± 0.28 ^a^	
Significant effects; *p*^treatment^ = 0.75			*p*^storage^ = 0.01	*p*^treatment^ × *p*^storage^ = 0.01	

Differences among storage periods are shown with small letters (*p* < 0.05), differences among treatments are shown with capital letters (*p* < 0.05).

**Table 4:** Pearson’s correlation coefficients between the measured parameters of *Agaricus bisporus* during the storage period.

	WL	EP	RR	L*	*C°*	*hue*	BI
WL	1						
EP	0.793	1					
RR	−0.830	0.319	1				
L*	−0.999 **	0.819	0.805	1			
*C^°^*	0.982 *	−0.888	−0.715	−0.988 *	1		
*hue*	0.992 **	−0.751	−0.854	−0.986 *	0.970 *	1	
BI	0.992 **	−0.865	−0.752	−0.996 **	0.998 **	0.978 *	1

*: Correlation is significant at *p* < 0.05. **: Correlation is significant at *p* < 0.01. Here, WL is weight loss, EP is ethylene production, RR is respiration rate, and BI is the browning index.

## Data Availability

All-new research data were presented in this contribution.

## References

[1] Tao F., Zhang M., Hangqing Y., Jincai S. (2006). Effects of different storage conditions on chemical and physical properties of white mushrooms after vacuum cooling. J. Food Eng..

[2] Singla R., Abhijit G., Ghosh M. (2010). Physicochemical and nutritional characteristics of organic acid-treated button mushrooms (*Agaricus bisporous*). Food Bioprocess Technol..

[3] Brennan M., Le Port G., Gormley R. (2000). Post-harvest treatment with citric acid or hydrogen peroxide to extend the shelf life of fresh sliced mushrooms. LWT-Food Sci. Technol..

[4] Jafri M., Jha A., Bunkar D.S., Ram R.C. (2013). Quality Retention of Oyster Mushrooms (*Pleurotus florida*) by a Combination of Chemical Treatments and Modified Atmosphere Packaging. Postharvest Biol. Technol..

[5] Khan Z.U., Bu J., Khan N.M., Khan R.U., Jiang Z., Mou W., Ying T. (2015). Integrated treatment of CaCl 2, citric acid and sorbitol reduces loss of quality of button mushroom (*Agaricus bisporus*) during postharvest storage. J. Food Process. Preserv..

[6] Çavuşoğlu Ş. (2018). Ambalajlama ve Metil Jasmonat Uygulamalarının *Pleurotus Ostreatus* (Jaqu. Ex Fr.) P. Kumm.(Oyster Mushroom) Mantarında Hasat Sonrası Kaliteye Etkileri. EJONS Int. J. Math. Eng. Nat. Sci..

[7] Gao M., Feng L., Jiang T. (2014). Browning inhibition and quality preservation of button mushroom (*Agaricus bisporus*) by essential oils fumigation treatment. Food Chem..

[8] Brennan M., Le Port G., Pulvirenti A., Gormley R. (1999). The effect of sodium metabisulphite on the whiteness and keeping quality of sliced mushrooms. LWT-Food Sci. Technol..

[9] Jiang T. (2013). Effect of Alginate Coating on Physicochemical and Sensory Qualities of Button Mushrooms (*Agaricus bisporus*) under a High Oxygen Modified Atmosphere. Postharvest Biol. Technol..

[10] Jiang T. (2012). Effect of Natamycin in Combination with Pure Oxygen Treatment on Postharvest Quality and Selected Enzyme Activities of Button Mushroom (*Agaricus bisporus*). J. Agric. Food Chem..

[11] Hu Y.H., Chen C.M., Xu L., Cui Y., Yu X.Y., Gao H.J., Wang Q., Liu K., Shi Y., Chen Q.X. (2015). Postharvest Application of 4-methoxy Cinnamic Acid for Extending the Shelf Life of Mushroom (*Agaricus bisporus*). Postharvest Biol. Technol..

[12] Lagnika C., Zhang M., Mothibe K.J. (2013). Effects of Ultrasound and High Pressure Argon on Physico-chemical Properties of White Mushrooms (*Agaricus bisporus*) during Postharvest Storage. Postharvest Biol. Technol..

[13] Wang Z., Chen L., Yang H., Wang A. (2015). Effect of Exogenous Glycine Betaine on Qualities of Button Mushrooms (*Agaricus bisporus*) during Postharvest Storage. Eur. Food Res. Technol..

[14] Nussinovitch A., Kampf N. (1993). Shelf life extension and conserved texture of alginate coated mushrooms (*Agaricus bisporus*). J. Food Technol..

[15] Mohebbi M., Ansarifar E., Hasanpour N., Amiryousefi M.R. (2012). Suitability of aloe vera and gum tragacanth as edible coatings for extending the shelf life of button mushroom. Food Bioprocess Technol..

[16] Sharma P., Kehinde B.A., Kaur S., Vyas P. (2019). Application of edible coatings on fresh and minimally processed fruits: A review. Nutr. Food Sci..

[17] Huang Q., Qian X., Jiang T., Zheng X. (2019). Effect of chitosan and guar gum based composite edible coating on quality of mushroom (*Lentinus edodes*) during postharvest storage. Sci. Hortic..

[18] Çavuşoğlu Ş., Keskin N., Türkoğlu N., Ozcan M., Demir H. (2019). Edible Coatings and Films and Its Use in Postharvest Cold Storage of Fruits.

[19] Nisperos-Carriedo M.O., Krochta J.M., Baldwin E.A., Nisperos-Carriedo M. (1994). Edible coatings and films based on polysaccharides. Edible Coatings and Films to Improve Food Quality.

[20] Gennadios A., McHugh T.H., Weller C.L., Krochta J.M., Krochta J.M., Baldwin E.A., Nisperos-Carriedo M. (1994). Edible coatings and films based on proteins. Edible Coatings and Films to Improve Food Quality.

[21] Krochta J.M., Gennadios A. (2002). Proteins as raw materials for films and coatings: Definitions, current status, and opportunities. Protein-Based Films and Coatings.

[22] Yousuf B., Srivastava A.K. (2015). Psyllium (Plantago) gum as an effective edible coating to improve quality and shelf life of fresh-cut papaya (*Carica papaya*). Int. J. Biol. Biomol. Agric. Food Biotechnol. Eng..

[23] Maqbool M., Ali A., Alderson P.G., Zahid N., Siddiqui Y. (2011). Effect of a novel edible composite coating based on gum arabic and chitosan on biochemical and physiological responses of banana fruits during cold storage. J. Agric. Food Chem..

[24] Vieira J.M., Flores-López M.L., de Rodríguez D.J., Sousa M.C., Vicente A.A., Martins J.T. (2016). Effect of chitosan-Aloe vera coating on postharvest quality of blueberry (*Vaccinium corymbosum*) fruit. Postharvest Biol. Technol..

[25] Karimirad R., Behnamian M., Dezhsetan S. (2019). Application of chitosan nanoparticles containing *Cuminum cyminum* oil as a delivery system for shelf life extension of *Agaricus bisporus*. LWT-Food Sci. Technol..

[26] Çavuşoğlu Ş. (2018). Effects of Modified Atmosphere and Methyl Jasmonate Treatments on The Postharvest Quality and Storage Life of Agaricus bisporus. J. Fungus.

[27] Bico S.L.S., Raposo M.F.J., Morais R.M.S.C., Morais A.M.M.B. (2009). Combined effects of chemical dip and/or carrageenan coating and/or controlled atmosphere on quality of fresh-cut banana. Food Control..

[28] Ziedan E.S.H., El Zahaby H.M., Maswada H.F., Zoeir E.H.A.E.R. (2018). Agar-agar a promising edible coating agent for management of postharvest diseases and improving banana fruit quality. J. Plant Prot. Res..

[29] Zhu D., Guo R., Li W., Song J., Cheng F. (2019). Improved postharvest preservation effects of Pholiota nameko mushroom by sodium alginate–based edible composite coating. Food Bioprocess Technol..

[30] Tarlak F., Ozdemir M., Melikoglu M. (2020). The combined effect of exposure time to sodium chlorite (NaClO2) solution and packaging on postharvest quality of white button mushroom (*Agaricus bisporus*) stored at 4 °C. Food Sci. Technol..

[31] Mohapatra D., Frias J.M., Oliveira F.A.R., Bira Z.M., Kerry J. (2008). Development and validation of a model to predict enzymatic activity during storage of cultivated mushrooms (*Agaricus bisporus* spp.). J. Food Eng..

[32] Jolivet S., Arpin N., Wicher H.J., Pellon G. (1998). Agaricus bisporus browning: A review. Mycol Res..

[33] Vamos-Vigyazo L. (1981). Polyphenol oxidases and peroxidases in fruits and vegetables. CRC Crit. Rev. Food Sci. Nutr..

[34] Oms-Oliu G., Aguiló-Aguayo I., Martín-Belloso O., Soliva-Fortuny R. (2010). Effects of pulsed light treatments on quality and antioxidant properties of fresh-cut mushrooms (*Agaricus bisporus*). Postharvest Biol. Technol..

[35] Pen L.T., Jiang Y.M. (2003). Effects of chitosan coating on shelf life and quality of fresh-cut Chinese water chestnut. LWT-Food Sci. Technol..

[36] Mohammadı L., Khankahdanı H.H., Tanaka F., Tanaka F. (2021). Postharvest Shelf-life Extension of Button Mushroom (*Agaricus bisporus* L.) by Aloe vera Gel Coating Enriched with Basil Essential Oil. Environ. Control. Biol..

[37] Jiang Y.M., Yao L., Lichter A., Li J.R. (2003). Postharvest biology and technology of litchi fruit. J. Food Agric. Environ..

[38] Yang Z., Cao S., Su X., Jiang Y. (2014). Respiratory activity and mitochondrial membrane associated with fruit senescence in postharvest peaches in response to UV-C treatment. Food Chem..

[39] Han Q., Gao H., Chen H., Fang X., Wu W. (2017). Precooling and ozone treatments affects postharvest quality of black mulberry (*Morus nigra*) fruits. Food Chem..

[40] Ozdemir A.E., Didin O., Candir E., Kaplankiran M., Yildiz E. (2019). Effects of rootstocks on storage performance of Nova mandarins. Turk. J. Agric. For..

[41] Koyuncu M.A. (2020). The effect of hot water, 1-MCP, and lovastatin on fresh-cut apples after long-term controlled atmosphere storage. Turk. J. Agric. For..

[42] Nasiri M., Barzegar M., Sahari M., Niakousari M. (2018). Application of tragacanth gum impregnated with Satureja khuzistanica essential oil as a natural coating for enhancement of postharvest quality and shelf life of button mushroom (*Agaricus bisporus*). Int. J. Biol. Macromol..

[43] Çavuşoğlu Ş., Gökçenay G. (2018). Farklı dozlarda uygulanan sitokininin beyaz şapkalı mantarın (*Agaricus bisporus*) muhafazası üzerine etkisi. Mantar Derg..

[44] Akan S., Horzum Ö (2020). Use of modified atmosphere packaging to manage quality of green garlic leaves during cold storage period. Emir. J. Food Agric..

[45] Xu S., Chen X., Sun D.W. (2001). Preservation of kiwifruit coated with an edible film at ambient temperature. J. Food Eng..

[46] Li H., Yu T. (2001). Effect of chitosan on incidence of brown rot, quality and physiological attributes of postharvest peach fruit. J. Sci. Food Agric..

[47] Tesfay S.Z., Magwaza L.S., Mbili N., Mditshwa A. (2017). Carboxyl methylcellulose (CMC) containing moringa plant extracts as new postharvest organic edible coating for Avocado (*Persea americana* Mill.) fruit. Sci. Hortic..

